# Combining in vitro and in vivo screening to identify efficient *Pseudomonas* biocontrol strains against the phytopathogenic bacterium *Ralstonia solanacearum*

**DOI:** 10.1002/mbo3.1283

**Published:** 2022-04-14

**Authors:** Sophie E. Clough, Alexandre Jousset, John G. Elphinstone, Ville‐Petri Friman

**Affiliations:** ^1^ Department of Biology University of York York UK; ^2^ Department of Biosciences Chemistry Durham University Durham UK; ^3^ Department of Biology, Institute of Environmental Biology, Ecology and Biodiversity Group Utrecht University Utrecht The Netherlands; ^4^ Fera Science Ltd. York UK

**Keywords:** environmental microbiology, microbial ecology, microbial interactions and pathogenesis, plant–microbe interactions

## Abstract

Although plant pathogens are traditionally controlled using synthetic agrochemicals, the availability of commercial bactericides is still limited. One potential control strategy could be the use of plant growth‐promoting bacteria (PGPB) to suppress pathogens via resource competition or the production of antimicrobial compounds. This study aimed to conduct in vitro and in vivo screening of eight *Pseudomonas* strains against *Ralstonia solanacearum* (the causative agent of bacterial wilt) and to investigate underlying mechanisms of potential pathogen suppression. We found that inhibitory effects were *Pseudomonas* strain‐specific, with strain CHA0 showing the highest pathogen suppression. Genomic screening identified 2,4‐diacetylphloroglucinol, pyoluteorin, and orfamides A and B secondary metabolite clusters in the genomes of the most inhibitory strains, which were investigated further. Although all these compounds suppressed *R. solanacearum* growth, only orfamide A was produced in the growth media based on mass spectrometry. Moreover, orfamide variants extracted from *Pseudomonas* cultures showed high pathogen suppression. Using the “Micro‐Tom” tomato cultivar, it was found that CHA0 could reduce bacterial wilt disease incidence with one of the two tested pathogen strains. Together, these findings suggest that a better understanding of *Pseudomonas*–*Ralstonia* interactions in the rhizosphere is required to successfully translate in vitro findings into agricultural applications.

## Graphical abstract

This study aimed to conduct in vitro and in vivo screening of eight *Pseudomonas* strains against *Ralstonia solanacearum* (the causative agent of bacterial wilt) and to investigate underlying mechanisms of potential pathogen suppression. Genomic screening identified 2,4‐diacetylphloroglucinol, pyoluteorin, and orfamides A and B secondary metabolite clusters in the genomes of the most inhibitory strains, which all suppressed *R. solanacearum* growth. Although *Pseudomonas* could reduce bacterial wilt disease incidence with tomato, the effect depended on the *R. solanacearum* strain, highlighting the need to better understand *Pseudomonas*–*Ralstonia* interactions in the rhizosphere.

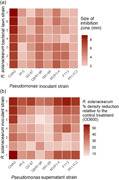

## 1 INTRODUCTION

The use of chemical pesticides has declined in recent years due to elevated costs, environmental toxicity, and stricter legislation (Chen et al., 2016). As a result, new methods and approaches are required to control plant pathogens and to ensure future food security in the face of expanding human population (Hayward, 1991; Kaczmarek et al., 2014; Wang et al., 2019). One alternative to traditional agrochemicals is biocontrol, which depends on using natural microbial competitors against a variety of pathogens to restrict pathogens' growth and survival. Several biocontrol agents have been identified and found to show a broad range of activity against bacterial, fungal, viral, and nematode pathogens (Hansen & Keinath, 2013; Nagachandrabose, 2020; Sofrata et al., 2011; Wang et al., 2019). Plant growth‐promoting bacteria (PGPB) are one example of potentially highly effective biocontrol agents, with a broad‐range biocontrol activity (Pierson et al., 1998). PGPB biocontrol outcomes are, however, often variable in field conditions, despite clear inhibition observed under laboratory conditions (Compant et al., 2005). One reason for this is that lab conditions fail to replicate actual field conditions in terms of nutrient availability, competition with rhizospheric bacteria, degradation, and adsorption of secreted antimicrobials, and the likelihood of successful colonization in natural environments, these can all affect the efficacy of disease suppression (Weller, 2007). In addition, even though biocontrol agents can show a broad range of effects against different types of pathogens, they might not be able to inhibit different genotypes of one given pathogen species (Xue et al., 2013). Considering pathogen‐biocontrol agent interactions at the genotype level, and the mechanisms by which inhibition is mediated in more realistic environmental conditions, are therefore required to develop functionally robust biocontrol methods.

Plant pathogenic bacteria belonging to the *Ralstonia solanacearum* species complex (RSSC) cause bacterial wilt disease in many wild and cultivated plants. It is ranked the second most important bacterial plant pathogen globally and possesses numerous virulence mechanisms that allow it to infect more than 250 plant species from 54 plant families (Genin & Denny, 2012; Hayward, 1991; Mansfield et al., 2012; Nion & Toyota, 2015). RSSC has spread globally across the world and has a quarantine status in many countries meaning it is monitored regularly to prevent further spread (EPPO, 2021). In addition to the trade of infected plant materials, RSSC global distribution is likely explained by its high genetic variability and ability to rapidly adapt to surrounding environmental conditions (Genin, 2010; Genin & Denny, 2012). Between‐strain differences include variation in pathogenicity‐related genes, such as the presence of Type III secretion effectors and genes involved in chemotaxis, adherence, secondary metabolic pathways, biosynthesis of phytohormones, and the detoxification of various antimicrobial compounds which are important in determining *R. solanacearum* host range (Genin, 2010; Genin & Denny, 2012). This variation can be generated via accumulation of mutations (Gopalan‐Nair et al., 2021; Guidot et al., 2014) or horizontal gene transfer between different *R. solanacearum* strains, which can result in an exchange of virulence traits between different genotypes (Guidot et al., 2009). Although *R. solanacearum* strains in Europe are considered to be clonal, belonging to the phylotype IIB group (Castillo & Greenberg, 2007; Clarke et al., 2015), some strain variation exists at the local scale within countries (Caruso et al., 2017; Parkinson et al., 2013). To what extent this variation affects the pathogen response to biocontrol PGPBs is, however, currently unknown.

Traditionally, the development of a biocontrol method begins in vitro in the lab by identifying bacterial strains with potential biocontrol activity (Fravel, 2005). Biocontrol efficiency within *Pseudomonas* plant growth‐promoting species is often mediated by a variety of secreted secondary metabolites, including several antimicrobials and iron‐scavenging siderophores (Becker et al., 2012). Some biocontrol characteristics are more common amongst these strains, including the ability to produce 2,4‐diacetylphloroglucinol (DAPG) and hydrogen cyanide—which are effective antimicrobial against bacterial, fungal, and nematode plant pathogens (Almario et al., 2017; Compant et al., 2005; Cronin et al., 1997; Haas & Défago, 2005; Haas & Keel, 2003; Humair et al., 2009). In contrast, other biocontrol properties are strain‐specific, such as the production of cyclic orfamide lipopeptides by certain subgroups of *Pseudomonas fluorescens* such as *Pseudomonas protegens* strains: CHA0 and Pf‐5 (Ma et al., 2016). Methods for screening bacterial biocontrol agents against *R. solanacearum* include exposing pathogenic bacterial strains to biocontrol bacteria directly in co‐cultures and measuring pathogen growth or survival as a function of time (Fravel, 2005). Supernatant assays are also often used to test if secretions in the media suppress the growth of the pathogens (Kaur et al., 2019; C. Yang et al., 2019). However, the successful identification of biocontrol strains in vitro does not always translate to successful biocontrol outcomes in vivo and *Pseudomonas* biocontrol outcomes are often variable in field conditions despite clear inhibition observed in laboratory conditions. For example, *Pseudomonas* strains have been shown to unsuccessfully replicate in vitro protection against *R. solanacearum* during in vivo experiments with *Eucalyptus* trees, and it was suspected this may have been due to low‐level expression of genes responsible for biocontrol activity (Ran et al., 2005). The success of biocontrol is further influenced by competition with native bacteria in the rhizosphere, and it has also been shown that the effectiveness of their secondary metabolites, such as DAPG, can be influenced by the age and species of the host plant is has been selected to protect (Notz et al., 2001; Siddiqui & Shaukat, 2003). Soil properties such as moisture, temperature, clay content, and pH can also influence the success of biocontrol (van der Putten et al., 2006), while common bacterial feeders, such as protozoa and nematodes, could also predate the biocontrol bacteria (Pedersen et al., 2009). Therefore, it is vital that in vitro screening is coupled with in vivo validation of biocontrol effectiveness with plants to develop successful biocontrol applications with translational potential.

Here we used such an approach to screen and identify efficient *Pseudomonas* biocontrol bacterial strains against six UK and one Polish *R. solanacearum* isolates that belong to an economically important potato‐specific RSSC lineage (Phylotype IIB sequevar 1; formerly known as race3 biovar2 strains). We first tested direct and indirect interactions between *R. solanacearum* strains and eight plant growth‐promoting *Pseudomonas* strains in vitro, which were chosen for their previously characterized antimicrobial activity against a Chinese *R. solanacearum* isolate belonging to Phylotype I (Hu et al., 2016). We then conducted genomic screening of *Pseudomonas* strains to identify potential antimicrobial secondary metabolite clusters and directly tested whether these compounds were produced and if they inhibited *R. solanacearum* under laboratory conditions. Finally, the biocontrol potential of the *P. protegens* CHA0 strain was tested against two *R. solanacearum* strains in the tomato rhizosphere. Results revealed that antimicrobial effects of *Pseudomonas* were strain‐specific, and the strongest inhibitory effects were mediated by *P. protegens* strain CHA0. Of the tested compounds, the pyoluteorin antibiotic had the strongest inhibitory effect followed by DAPG and orfamides A and B. While CHA0 also showed biocontrol efficiency with tomato, this was clear only with one of the tested UK *R. solanacearum* isolates. Together, these results show that while in vitro screening can be used to identify potential *Pseudomonas* biocontrol strains against *R. solanacearum*, more work is needed to understand their biocontrol activity in vivo in the plant rhizosphere.

## 2 METHODS

### 2.1 Culturing and maintenance of bacterial strains

Eight fluorescent Pseudomonad strains (CHA0, Pf‐5, Q2‐87, Q8R1‐96, 1M1‐96, MVP1‐4, F113, and Ph11C2) which have been studied extensively previously in biocontrol (Hu et al., 2016), and seven *Ralstonia solanacearum* strains (#1–#7), which were isolated as a part of annual river sampling survey in England and Wales by Fera Science Ltd., were used in the experiments (listed in Tables A1 and A2). All bacteria were stored in 20% glycerol stocks at −80°C. Before experiments, bacterial starting cultures were prepared as follows: frozen samples were inoculated in 5 ml of LB, NB, or CPG broth (media recipes described in Table A3) and incubated with shaking at 200 rpm at 28°C for 24 h. Rich media preparations were used throughout all experiments to ensure the efficient growth of both bacterial genera. Bacterial cultures were prepared similarly throughout all experiments unless stated otherwise.

### 2.2 Measuring *R. solanacearum* inhibition by *Pseudomonas* strains in direct contact

**Figure A1:**
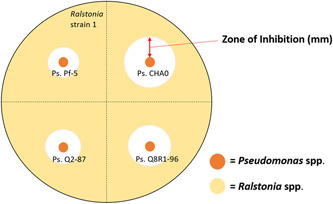
Experimental design quantifying direct inhibition of *Ralstonia solanacearum* strain #1 by four types of *Pseudomonas* strains using soft agar overlay assay

Soft agar overlay assays were used to test direct inhibition of *R. solanacearum* by *Pseudomonas* strains. *Pseudomonas* strains were grown for 24 h in 5 ml of 100% LB broth at 28°C with shaking at 200 rpm and *R. solanacearum* strains were grown for 30 h under the same conditions*.* Soft agar overlay plates were prepared by first filling sterile 90‐mm Petri dishes with a layer of hard LB which was left to solidify. Two hundred microliters of each *R. solanacearum* culture were then mixed with 20 ml of cooled (below 55°C) liquid soft agar and poured on top of the hard agar layer. As *R. solanacearum* grows more slowly than *Pseudomonas*, plates were left to incubate for ∼8 h before spotting the *Pseudomonas* cultures on top of the soft agar overlay as follows. Each plate was divided evenly into quarters and 2 μl of a *Pseudomonas* strain (∼1.0 × 10^6^ CFU/ml) was spotted on the center of each quarter of the plate (example shown in Figure A1). Plates were then incubated upside down at 28°C and zones of inhibition were recorded after 96 h. The distance of the inhibition zones was measured from the outer edge of the *Pseudomonas* spot to the *R. solanacearum* lawn (in millimeters with a ruler). Each strain combination treatment was carried out in triplicate.

### 2.3 Measuring *R. solanacearum* inhibition by *Pseudomonas* strains indirectly using supernatant assays

To investigate *R. solanacearum* inhibition by *Pseudomonas* in the absence of direct contact, we exposed *R. solanacearum* strains to supernatants of each *Pseudomonas* species in pairwise supernatant cultures. The *Pseudomonas* supernatants included all secondary metabolites excreted when *Pseudomonas* strains were grown alone in LB media and were prepared as follows. All *Pseudomonas* strains were first grown individually in 20 ml of LB broth (Table A3) for 24 h with shaking at 200 rpm. The supernatant was then prepared by centrifuging cultures for 10 min at 4000*g* before separating bacterial cells and fragments from soluble material including secondary metabolites using 0.2‐μm filters. The inhibition was measured using flat bottomed 96‐well plates in 50:50 *Pseudomonas* supernatant to LB mixtures. As a negative control *R. solanacearum* was grown in 50:50 LB in the sterile water mixture. At the start of the experiment, every supernatant mixture was inoculated with 2 μL of each *R. solanacearum* strain (∼1.0 × 10^6^ CFU/ml), and microplates were then incubated at 28°C for 3 days and their bacterial densities were recorded as optical density at 24 and 72 h (OD 600 nm; Tecan Infinite spectrophotometer). All pairwise combinations were replicated four times and control treatments three times.

### 2.4 Genome sequencing of *Pseudomonas* strains

Single colonies of each *Pseudomonas* strain were inoculated in NB broth and grown for 12 h at 30°C with shaking at 170 rpm. Genomic DNA was extracted using the QIAGEN blood and cell culture DNA kit (catalog No. QIAGEN Genomic‐tip 100/G, Midi 13343) following the manufacturer's protocol, and DNA quality was tested using nanodrop. All strains were sequenced using the Miseq platform (2 × 300 bp) at the Utrecht Sequencing Facilities. To obtain more accurate genome assemblies, reads were preprocessed as follows. We first removed adapter sequences, read shorter than 50 bp, and low‐quality nucleotides using a Phred quality score threshold of <20. Genome assemblies were carried out in two steps. First, we used SOAPdenovo v2.04 to assemble reads into contigs and scaffolds based on K‐mer size (available at https://soap.genomics.org.cn/). Second, GapCloser v1.12 was used to close gaps emerging during the scaffolding process by SOAPdenovo (Li et al., 2010).

### 2.5 Investigating *Pseudomonas* secondary metabolite biosynthesis gene clusters using antiSmash5.0

The de novo assembled draft genomes of the eight *Pseudomonas* strains were analyzed to identify potential secondary metabolic clusters linked with antibiosis using the antiSMASH 5.0 pipeline (https://antismash.secondarymetabolites.org; Blin et al., 2019, 2017), which allows rapid genome‐wide identification and in‐depth analysis of secondary metabolite biosynthesis gene clusters based on several open‐source databases. Fasta files were uploaded to antiSMASH5.0 bacterial version with detection strictness set to “relaxed” and all search features included to maximize potential metabolic clusters identified. Based on this analysis, we identified four antimicrobial compounds which could potentially be produced by our *Pseudomonas* strains. One of these compounds, DAPG, is a common antimicrobial produced by all eight fluorescent *Pseudomonas* strains, while three specific gene clusters for pyoluteorin (an antimicrobial with unknown mechanisms of action; Kidarsa et al., 2011) and orfamides A and B (cyclic lipopeptides that can cause membrane pore formation; Ma et al., 2016) were found only in CHA0 and Pf‐5 *Pseudomonas* genomes.

### 2.6 Determining the effects of identified *Pseudomonas* metabolites on *R. solanacearum* growth in single‐compound and multicompound mixtures

Pure compounds of orfamide A (Santa Cruz Biotechnology), orfamide B (Santa Cruz Biotechnology), Pyoluteorin (Santa Cruz Biotechnology), and DAPG (Santa Cruz Biotechnology) were purchased to test their efficacy against *R. solanacearum* strains. Ten millimolar stocks were made in 100% dimethyl sulfoxide (DMSO) and stored at −20°C, except for DAPG, for which a 100 mM stock was prepared in 100% methanol and stored at −20°C. The effect of DAPG was measured by inoculating 2 μl of each *R. solanacearum* strain (∼1.0 × 10^6^ CFU/ml) to 100% LB broth with DAPG at the following concentrations: 1000, 500, 100, 50, and 0 μM (negative control). Due to the relatively high costs of chemicals, only two *R. solanacearum* strains (#1 and #7) were tested for their susceptibility to pyoluteorin (100 μM concentration only). Similarly, only Strain #1 susceptibility was tested to orfamides A and B (100 μM concentration only) following the same methods as with DAPG. Bacterial densities were recorded as optical density at 0, 24, 48, and 72 h after inoculation with a spectrophotometer (OD 600 nm; Tecan Sunrise spectrophotometer).

### 2.7 Confirmation of the production of secondary metabolites in the *Pseudomonas* supernatant using mass spectrometry

Mass spectrometry was used to identify and quantify any secondary metabolites produced by CHA0 and Pf‐5 in the growth conditions they were exposed to in vitro (compounds were identified against chemical standards). We also used untargeted analysis to identify any novel antimicrobials produced by comparing peaks against existing databases.

The strains were grown in triplicate in LB broth with 200 rpm shaking at 28°C for 24 h. Following incubation, bacterial densities were normalized to an optical density of 0.1 (OD 600 nm), and the cultures were centrifuged for 10 min at 4000*g.* The supernatant was then filtered using 0.2‐μm filters to separate bacterial cells and fragments from secondary metabolites present in the supernatants. A total of six samples were produced (two *Pseudomonas* strains in one growth condition in triplicate). Two hundred microliters of each sample were provided to the Centre of Excellence in Mass Spectrometry at the University of York for mass spectrometry analysis. The LC separation was performed by an Acquity UPLC I class system (Waters), on a BEH C18 100 × 2.1, 1.7 U column (Waters). The sample injection volume was 7.5 µl. The MS end was a Synapt G2S‐Si QTOF (Waters) mass spectrometer which was operated in positive ESI, resolution mode, using the HDMS^E^ acquisition technique (alternating scans of MS and MS^2^ acquisitions [fragmentation by CID] alongside traveling wave ion mobility separation). Voltages in the low energy function (MS) were 4 V both in the trap and transfer cell; and in the high energy function (MS^2^), the voltage was 4 V in the trap and a 20–100 V ramp in the transfer cell. The source was operated with the capillary set to 3 kV, source temperature 150°C, desolvation temperature 450°C, cone gas flow 20 L/h, desolvation gas flow 450 L/h, and nebulizer 6.5 bar. Trap gas flow was 2 ml/min, helium cell flow 180 ml/min, and IMS gas flow 80 ml/min. Mass range was 100–1000 *m*/*z*, scan time 0.2 s, lock mass was leucine enkephalin at *m*/*z* 556.2771.

Data was collected using MassLynx Software Version V4.2 SNC983 (Waters) and analyzed using UNIFI 1.9 (Waters) for semi‐quantitative analysis of target analytes (orfamide A, orfamide B, pyoluteorin, and DAPG). Calibration curves were constructed for target analytes at 10, 50, 100, 300, and 500 nM in LB media; the MS response for all analytes was linear in this range. Analytes were quantified using the calibration curve in the appropriate medium. Blanks, standards, and samples were injected in technical triplicates from the same vial. Progenesis QI v.2.0 (Waters) was used for the untargeted analysis of metabolomics data: putative compound identification was based on a score comprising MS match of exact mass of the compound precursor (ChemSpider database search with 10 ppm mass tolerance) and a match to in silico MS^2^ fragmentation pattern. Further data processing and downstream analysis were performed using Bioconductor package XCMS in R, combined with a database (LipidMaps) search.

Untargeted analyses were also conducted by comparing the collected dataset with the Knapsack database (https://www.knapsackfamily.com/KNApSAcK/). Briefly, Waters.raw files were converted to zlib compressed 32‐bit precision.mzML files using ProteoWizard MSConvert [1] version 3.0.19172, using the combineIonMobilitySpectra filter, and spectra centroided using the qtofpeakpicker with resolution set to 20,000 and threshold 1. Using custom scripts in R 3.0.0 operating in a Linux 64‐bit environment.mzML files were further processed using the mzR package [1] to lockmass correct spectra against leucine enkephalin ([M + H]^+^ = 556.27568, [M − H]^−^ = 554.26202). Lockmasses were identified within a ±0.1 Da *m*/*z* window, and the running mean of lockmasses in a 60 s moving window was used to adjust the *m*/*z* values for every scan. Across files, feature detection was achieved using the xcmsSet() function from the xcms package [2], using the following parameters: method = ‘centWaveWithPredictedIsotopeROIs’, ppm = 10, snthresh = 10, peakwidth = c(3, 20), prefilter = c(3, 1000), integrate = 2, mzdiff = −0.1, firstBaselineCheck = FALSE. Custom scripts were used to retain Gaussian features, which were then aligned and missing values recalculated across samples using the xcms group() and fillPeaks() functions, respectively. Feature areas were adjusted by subtracting the mean + 3 × the standard deviation of blank samples, with adjusted values <0 being set to 0. The feature list was then filtered to retain only the most intense monoisotope belonging to a single compound as identified by CAMERA [3], with areas above 0 in at least 90% of blank‐subtracted samples. These filtered features were reported as masstags (unique *m*/*z* and retention time pairs) and annotated where possible against authentic standards or putative compounds from literature or databases based on exact mass. The Progenesis QI v.2.0 (Waters) was also used to search for compounds by their monoisotopic mass which has been reported to be present in *Pseudomonas* genomes from the literature. The potential metabolites were identified if their observed monoisotopic mass matched with the accurate known ppm within a range of ±10 ppm. However, without a chemically verified standard for definitive identity matches, this can only be considered speculative.

### 2.8 Extraction and isolation of *Pseudomonas* orfamide antimicrobials and testing their effects on *R. solanacearum* growth

A protocol was adapted from Ma et al. (2016) to extract cyclic lipopeptide orfamides from the CHA0 *Pseudomonas* strain. Briefly, a starting culture of CHA0 was grown in a 250 ml flask containing 50 ml of King's Broth growth medium at 28°C with shaking at 200 rpm. This was then inoculated into a 2 L flask containing 500 ml of liquid media and shaken at 150 rpm for 48 h. *Pseudomonas* supernatant was collected via centrifugation at 10,000*g* for 10 min (J25 XP series centrifuge). The supernatant was then acidified to a pH of 2.0 using 8 M HCl and stored overnight at 4°C. The precipitate was collected after centrifugation at 10,000*g* for 20 min and extracted with 1 ml of 100% methanol. At this stage in the centrifuge tube, there were two distinguishable precipitates, one at the bottom of the tube and one close to the top. It was decided to collect these separately and call them “A” and “B” as it was unknown if they would have different or similar properties. The organic phase was collected by spinning samples until dry in a vacuum concentrator (Savant svc SpeedVac 100 h concentrator) and samples were then dissolved in 300 μl of 100% DMSO to make orfamide variant stocks. Due to a limited amount of these orfamide variant stocks, only *R. solanacearum* strains #1 and #7 were used for inhibition testing. Inoculant cultures were grown in 5 ml of LB broth overnight and 2 μl of each *R. solanacearum* strain was inoculated with 1% orfamide variants in LB broth (200 μl final volume). Bacterial densities were recorded at 24, 48, and 72 h (OD 600 nm; Tecan Infinite spectrophotometer), and each treatment was carried out in triplicate.

### 2.9 Testing *Pseudomonas* biocontrol efficiency in vivo using a tomato plant model

To explore the translational potential of *Pseudomonas* CHA0 strain in vivo, we tested if any of the inhibitory effects observed in vitro could protect plants from *R. solanacearum* infections using the Micro‐Tom tomato cultivar. This cultivar of tomato was selected as it is well‐established and often used when studying *R. solanacearum* infections (Gu et al., 2016). The infectivity of two *R. solanacearum* UK strains (#1 and #7) was tested in the absence and presence of CHA0 (a negative water control treatment was included). Tomato (*Solanum lycopersicum* “Micro‐Tom”) plants were grown in an incubated light chamber at 28°C with 16:8 h light:dark conditions with regular watering. Seeds were sown in 7 cm seedling trays in 35 g of autoclaved compost (John Innes #2) where they remained for the entire experiment. Experiments were conducted between July and September 2019 in temperature‐controlled plant growth chambers (Sanyo MLR‐352) at the University of York. To test the biocontrol efficacy of CHA0, the *Pseudomonas* culture was prepared by inoculating 100 ml of CPG broth with 100 μl of frozen stock bacterial culture in 300 ml glass culture flasks and the strain was grown for 24 h at 28°C with shaking at 200 rpm. Bacterial culture was then washed of nutrient media by centrifuging at 4000*g* for 10 min and resuspending in sterile dH2O. Seeds were germinated and sown in 7 cm seedling trays with 35 g of autoclaved compost and regularly watered using sterile water (for growth conditions see earlier section). *Pseudomonas* strain was inoculated onto the soil of 4‐week old seedlings 1 week before *R. solanacearum* strains to allow time for the biocontrol bacteria to effectively colonize the soil and roots. Before inoculation, *Pseudomonas* cell densities were adjusted to an OD 600 nm of 0.25 (∼1.0 × 0^9^ CFU/ml) and 6 ml was poured into each pot, soaking through the soil and roots, and any excess culture remained in the saucer below. Roots of tomato seedlings were cut before *R. solanacearum* inoculation using a sterile scalpel to mimic natural pathogen entry points in the field. *R. solanacearum* strains #1 and #7 were cultured in 30 ml of CPG broth (Table A3) for 48 h with shaking at 200 rpm at 28°C and adjusted to an OD 600 nm of 0.25. A total of 1 ml of each *R. solanacearum* strain was inoculated at the base of the visible stems 1 week after *Pseudomonas* inoculation. Three replicates were used for each treatment and each replicate consisted of nine individual plants. All plants were watered 1 h before monitoring infection to be certain that wilting was not due to dehydration. Bacterial wilting symptoms were recorded daily by scoring the plants as “uninfected” or “infected” when plants showed clear wilting symptoms. The experiment was terminated 3 weeks after pathogen inoculation and above‐ground dry weight was recorded for each replicate plant.

### 2.10 Statistical analyses

We used repeated‐measures analysis of variance to analyze mean differences between pathogen densities for all experiments including temporal data such as *R. solanacearum* exposure to different secondary metabolites. Two‐way ANOVA was used to analyze the mean differences between treatments when only a one‐time point was used for analysis, followed by pairwise post hoc Tukey tests with 95% confidence levels and Bonferroni‐corrected *p* values. These analyses were used when interpreting the direct and indirect soft agar and bacterial supernatant interactions between *R. solanacearum* and *Pseudomonas*. Poisson glm models with *χ* ^2^ tests were used to analyze the binomial in vivo tomato infection data. All statistical analyses and graphs were produced using R (R Foundation for Statistical Computing, R Studio Version [3. 4. 4], Packages: ggplot, tidyverse, nlme, rcompanion).

## 3 RESULTS

### 3.1 Measuring the direct inhibition of *R. solanacearum* by *Pseudomonas* strains using soft agar assays

**Figure 1:**
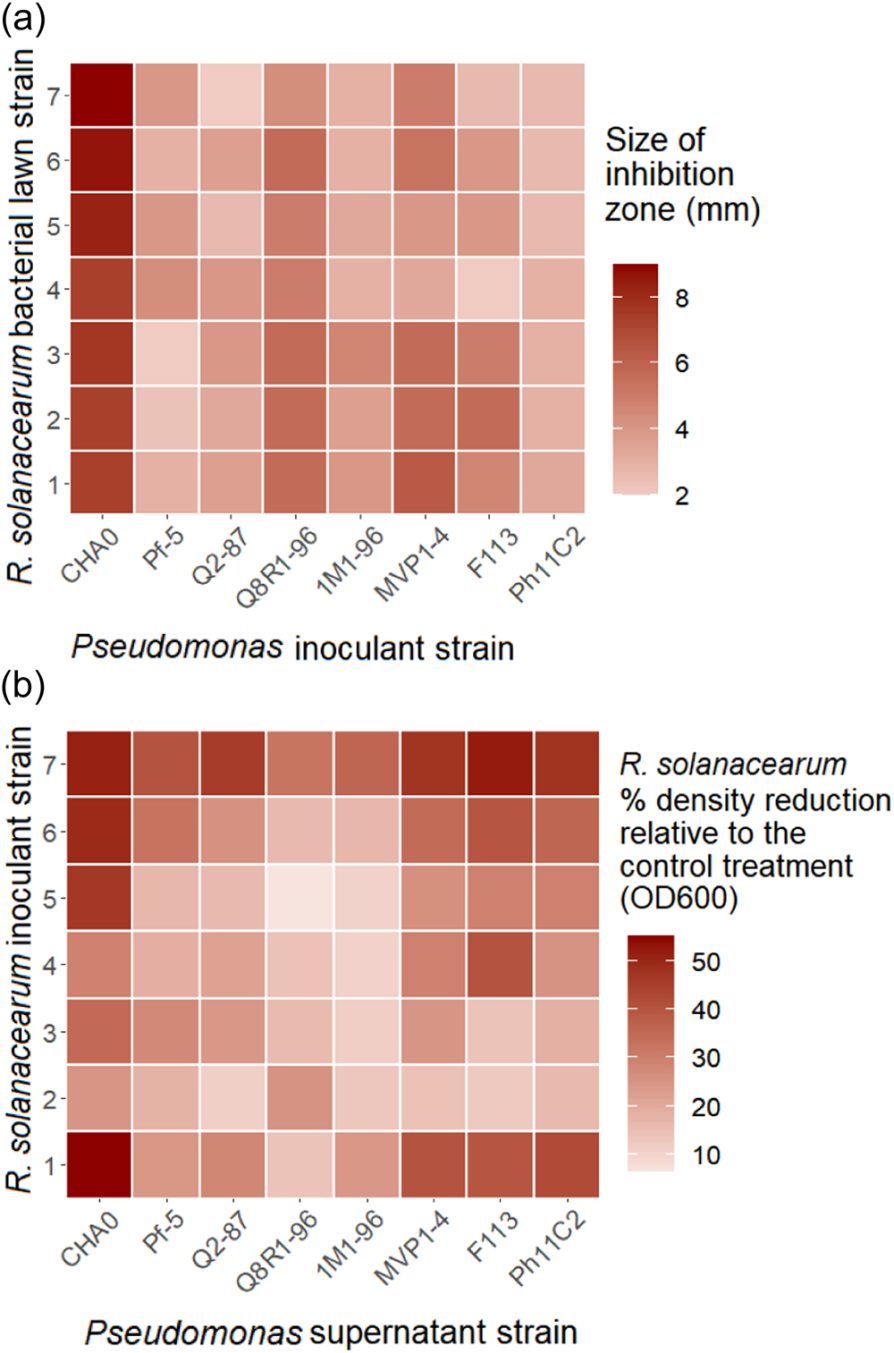
Direct and indirect inhibition of *Ralstonia solanacearum* by *Pseudomonas* strains. (a) Direct inhibition on soft agar assays, where each interaction represents a different *Pseudomonas* strain and the average diameter of the inhibition zones (mm) against different *R. solanacearum* strains after 96 h growth on soft agar lawns (*N* = 3). (b) Indirect inhibition of *R. solanacearum* by *Pseudomonas* strains' supernatant‐LB mixes (set up in 96‐well plates containing a 50:50 ratio of SN and 100% LB broth). The shade of red represents the growth reduction in *R. solanacearum* densities compared to the control (100% LB broth) treatment after 72 h of growth (*N* = 3)

Clear inhibition zones were detected in all pathogen strain treatments indicating that each *Pseudomonas* strain could inhibit the growth of *R. solanacearum* when in direct contact. However, inhibition zone sizes differed depending on the identities of interacting *Pseudomonas* and *R. solanacearum* strains (*Ralstonia* × *Pseudomonas*: *F*_42, 112_ = 3.877, *p* < 0.0001, Figure 1a). This variation appeared to be mainly driven by *Pseudomonas* strains, as post hoc analyses revealed only a small and nonsignificant variation between different *R. solanacearum* strains (*Ralstonia*: *F*_6, 161_ = 0.6364, *p* = 0.7009, Figure 1a). In contrast, much higher variation was observed between the inhibitory activity of *Pseudomonas* strains (*Pseudomonas*: *F*_7, 160_ = 60.81, *p* = 0.0001, Figure 1a), and post hoc analyses revealed that CHA0 was the most inhibitory strain, followed by Q8R1‐96 and MVP1‐4, which showed modest levels of inhibition (Tukey: *p* < 0.05). The strains 1M1‐96 and Ph11C2 caused the smallest zones of inhibition on *R. solanacearum* lawns overall (Tukey: *p* < 0.05). These results suggest that all *Pseudomonas* strains inhibited the growth of *R. solanacearum* in direct contact, which varied between different *Pseudomonas* strains.

### 3.2 Measuring the indirect inhibition of *R. solanacearum* by *Pseudomonas* strains using supernatant assays

Almost all *Pseudomonas* supernatants suppressed the growth of *R. solanacearum* compared to the no‐supernatant LB control treatments (*Supernatant treatment*: *F*_8, 236_ = 3.697, *p* = 0.01567, Figure 1b). In contrast to agar plate assays, *R. solanacearum* strain variation was more evident and the growth reduction of Strains #2 and #3 was significantly lower compared to other strains (*Ralstonia*: *F*_6, 217_ = 19.1102, *p* < 0.001, Figure 1b). The growth of Strains #1, #6, and #7 was reduced most by the *Pseudomonas* supernatants overall. In line with direct inhibition assays, the *Pseudomonas* CHA0 strain caused the greatest reduction in *R. solanacearum* growth, followed by strains Ph11C2 and MVP1‐4, which showed intermediate growth reduction (*Pseudomonas strain*: *F*_7, 216_ = 11.3595, *p* < 0.001, Figure 1b). *Pseudomonas* strains 1M1‐96 and Q8R1‐96 showed relatively small growth reduction. Together, these findings are qualitatively similar to the direct inhibition assay (Figure 1a), highlighting the high efficacy of the *Pseudomonas* CHA0 strain in suppressing the growth of *R. solanacearum*.

### 3.3 Comparative genomic analysis reveals variation in the presence of secondary metabolic clusters between different *Pseudomonas* strains

Comparative genomic analysis of secondary metabolite clusters based on the antiSMASH output identified between 11 and 17 metabolic clusters in each of the eight *Pseudomonas* genomes (Tables A4, A5, A6, A7, A8, A9, A10, A11). The type and diversity of recognized clusters are shown in Table 1. Nonribosomal peptide synthetases (NRPS) were the most abundant secondary metabolite clusters, which was expected as these account for several common antimicrobials produced by fluorescent *Pseudomonas* strains (Girard et al., 2020; Hesse et al., 2018). Similarly, DAPG metabolite (belonging to the T3PKS cluster), as well as the pyoverdine siderophore (NRPS cluster) metabolite clusters, were found in all strains. Overall, the highest number of clusters were detected in CHA0 and Pf‐5 strains (17). These strains also harbored some unique metabolite clusters such as the T1PKS metabolic cluster, which encodes pyoluteorin antimicrobial, and the CDPS cluster, which encodes unknown metabolites. CHA0 and Pf‐5 also had the greatest number of NRPS clusters and were the only two strains capable of producing the cyclic lipopeptides known as orfamides. Other *Pseudomonas* strains also possessed some unique clusters such as the ectoine metabolic cluster found in the Q2‐87 strain. A more in‐depth insight into the clusters and the percentage similarity with various *Pseudomonas* and other bacterial genomes based on the antiSMASH database is shown in Tables A4, A5, A6, A7, A8, A9, A10, A11. The all clusters which were investigated further had 95%–100% similarity with already characterized secondary metabolite clusters, increasing the certainty of their predicted identity and functioning.

**Table 1:** antiSMASH5.0 cluster types recognized in eight *Pseudomonas* genomes used in this study including a brief definition of their function

*Pseudomonas* strain	Total no. of clusters	Cluster types	Description of metabolic clusters and examples of metabolites
CHA0	17	NRPS (8), Bacteriocin (2), CDPS (1), T1PKS (1), T3PKS (1), NAGGN (1), Arylpolyene (1), Butyrolactone (1), Other (1)	–NRPS (nonribosomal peptide synthetase cluster) e.g., orfamide, lipopeptide, viscosin, rhizomide, pyoverdine, enantiopyochelin –NRPS‐like (NRPS‐like fragment) e.g., mangotoxin, lankacidin –Bacteriocin (unspecified ribosomally synthesized and posttranslationally modified peptide product [RiPP] cluster) e.g., unknown –CDPS (tRNA‐dependent cyclodipeptide synthases) e.g., unknown –T1PKS (type I polyketide synthase) e.g., pyoluteorin –T3PKS (type III polyketide synthase) e.g., unknown –NAGGN (N‐acetylglutaminylglutamine amide) e.g., unknown –Arylpolyene (arylpolyene cluster) e.g., APE Vf –Butyrolactone (butyrolactone cluster) e.g., unknown –Betalactone (beta‐lactone containing protease inhibitor) –Ectoine (ectoine cluster) e.g., unknown –Lanthipeptide (lanthipeptide cluster) e.g., putative class II –LAP (linear azol(in)e‐containing peptides e.g., unknown –Other (unknown) e.g., pyrrolnitrin
Pf‐5	17	NRPS (7), NRPS‐like (1), Bacteriocin (2), CDPS (1), T1PKS (1), T3PKS (1), NAGGN (1), Arylpolyene (1), Betalactone (1), Other (1)	
Q2‐87	15	NRPS (4), NRPS‐like (1), Bacteriocin (3), T3PKS (1), NAGGN (1), Arylpolyene (1), Butyrolactone (1), Betalactone (1), Ectoine (1), Lanthipeptide (1)	
Q8R1‐96	13	NRPS (6), NRPS‐like (1), Bacteriocin (1), T3PKS (1), NAGGN (1), Arylpolyene (1), Butyrolactone (1), Betalactone (1)	
1M1‐96	12	NRPS (3), NRPS‐like (1), Bacteriocin (1), T3PKS (1), NAGGN (1), Arylpolyene (1), Butyrolactone (1), Betalactone (1), Lanthipeptide (2)	
MVP1‐4	13	NRPS (4), NRPS‐like (1), Bacteriocin (1), T3PKS (1), NAGGN (1), Arylpolyene (1), Butyrolactone (1), Betalactone (1), Lanthipeptide (2), LAP (1)	
F113	11	NRPS (2), NRPS‐like (2), Bacteriocin (1), T3PKS (1), NAGGN (1), Arylpolyene (1), Butyrolactone (1), Betalactone (1), Lanthipeptide (1)	
Ph11C2	14	NRPS (4), NRPS‐like (1), Bacteriocin (3), T3PKS (1), NAGGN (1), Arylpolyene (1), Butyrolactone (1), Betalactone (1), Lanthipeptide (1)	

### 3.4 Confirming the production of *Pseudomonas* secondary metabolites using mass spectrometry

**Figure A2:**
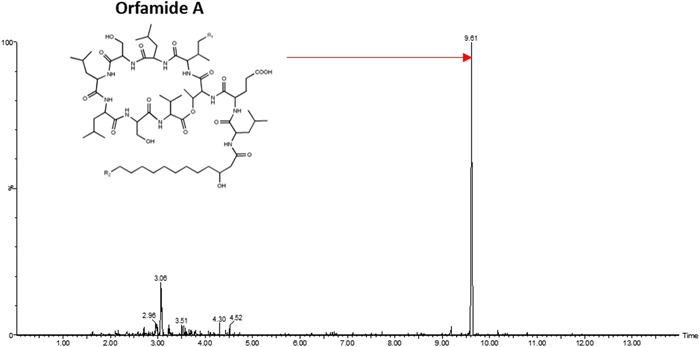
Mass spectrometry analysis indicating the presence of *Pseudomonas* metabolite orfamide A in the supernatant of strain CHA0. Orfamide A is identified with a monoisotopic mass (*m*/*z*) of 1317.826 [M + Na]^+^ and a retention time (*t*_R_) of 9.61 s at 15 ppm. This spectrum represents the replicate with the strongest peak out of the three replicates

Our genome screening results suggest that *Pseudomonas* inhibitory activity could have been due to the presence of DAPG or certain, less common, secondary metabolism clusters encoding pyoluteorin and Orfamides that were identified only in CHA0 and Pf‐5 strains. Due to the presence of these two unique clusters, CHA0 and Pf‐5 strains were chosen for a more detailed study. To verify the production of these compounds in LB media, the supernatants of CHA0 and Pf‐5 were analyzed using mass spectrometry against chemical standards for DAPG, pyoluteorin, orfamide A, and orfamide B. Based on matching monoisotopic masses to a database of *Pseudomonas* metabolites, a list of putative metabolites present in the *Pseudomonas* supernatants of CHA0 and Pf‐5 was determined using Progenesis QI v.2.0 (Waters) analysis (Table A12). These included compounds such as enantiopyochelin, rhizoxin, and indole‐3‐acetic acid which have different metabolic functions that can enhance bacterial survival (Table A12). For example, pyochelin is a siderophore that can aid iron acquisition when *Pseudomonas* may be in an iron‐limited environment to improve its survival chances and outcompete other bacterial competitors (Duffy & Defago, 1999)*.* Based on a comparison with four available standards, only orfamide A production by CHA0 strain was detected in LB supernatant with an average concentration of 7.5 mg/ml (Figure A2). In contrast, none of the other four candidate compounds were detected in Pf‐5 supernatant in LB media. These results suggest that only orfamide A was produced by CHA0 in the conditions used in the supernatant inhibition assays. However, approximately 70 unidentified metabolites were detected in the samples, which could also have contributed to *R. solanacearum* inhibition.

### 3.5 Testing the inhibitory effects of identified *Pseudomonas* secondary metabolites on *R. solanacearum* growth

**Figure 2:**
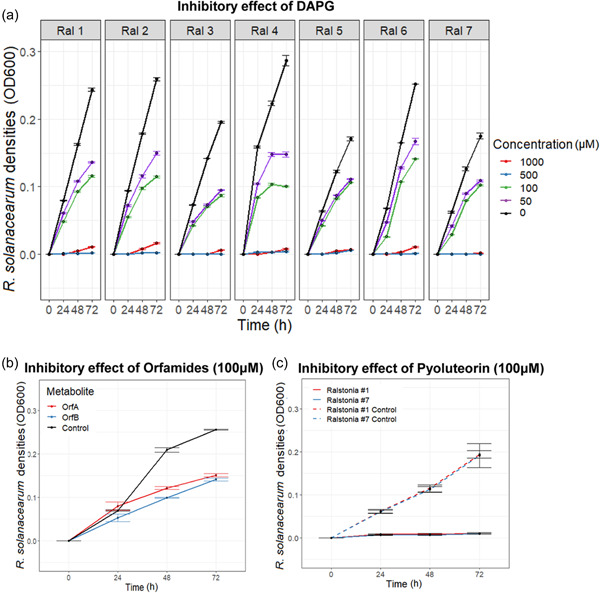
Testing the inhibitory effects of identified *Pseudomonas* secondary metabolites on *Ralstonia solanacearum* growth. Panel (a) shows the growth of seven *R. solanacearum* strains (in different panels) in various DAPG concentrations over time (1000 μM—red, 500 μM—blue, 100 μM—green, 50 μM—purple, and 0 μM LB broth [0 μM—control]—black). Panel (b) shows *R. solanacearum* strain #1 growth in the absence and presence of 100 μM of orfamides A and B. Panel (c) shows the growth of *R. solanacearum* strains #1 and #7 in the absence and presence of 100 μM of pyoluteorin. In all panels, bars show the standard error of the mean (±1 SEM) based on three replicates

The inhibitory effects of DAPG, pyoluteorin, and orfamides A and B were tested individually against *R. solanacearum* strains using commercially available chemical standards. The effect of DAPG was tested against all *R. solanacearum* strains, while the effect of orfamides and pyoluteorin were tested against a subset of strains due to the high costs of chemical standards (*Ralstonia* strain #1 for orfamides, and *R. solanacearum* strains #1 and #7 for pyoluteorin). It was found that DAPG suppressed every *R. solanacearum* strain in a concentration‐dependent manner (*Concentration*: *F*_4, 200_ = 238.2599, *p* < 0.001, Figure 2a), and all tested strains were unable to grow at the two highest concentrations (500 and 1000 μM). When all concentrations were included in the analysis, the effect of DAPG was independent of the *R. solanacearum* strain identity (*Ralstonia*: *F*_6, 98_ = 0.724, *p* = 0.6305, Figure 2a). However, excluding the two highest concentrations, where all growth was inhibited, some *R. solanacearum* strain differences were revealed (*Ralstonia*: *F*_6, 56_ = 3.355, *p* = 0.0068, Figure 2a): Strain #4 was the least susceptible to DAPG, while strains #3, #5, and #7 were relatively more susceptible. We also considered growth reduction by DAPG with growth in the absence of DAPG at the final time point for all *R. solanacearum* strains. Growth reduction was greater in 100 μM DAPG than 50 μM (*Concentration*: *F*_1, 124_ = 46.8, *p* < 0.001, Figure 2a) and between strain variation was evident at both concentrations (*Ralstonia* at 100 μM: *F*_6, 56_ = 5.671, *p* < 0.001, Figure 2a; *Ralstonia* at 50 μM: *F*_6, 56_ = 3.856, *p* = 0.00273, Figure 2a). Post hoc analyses revealed that Strains #5 and #6 were the least susceptible to DAPG, while Strains #2 and #3 were relatively more susceptible. Both orfamides A and B reduced the growth of *R. solanacearum* strain #1 and this effect became clearer over time (*Metabolite: F*_2, 6_ = 460.7, *p* < 0.001; *Metabolite* × *Time*: *F*_4, 12_ = 45.806, *p* = 0.0359, Figure 2b). However, no difference was found between orfamides A and B (*p* = 0.11). No visible growth was observed when either *R. solanacearum* strains #1 or #7 were exposed to pyoluteorin (*Ralstonia*: *F*_2, 16_ = 48.596, *p* < 0.001, Figure 2c), suggesting that both strains were highly susceptible to this compound.

### 3.6 Testing the inhibitory effects of orfamide variants isolated from *Pseudomonas* CHA0 on *R. solanacearum* growth

**Figure 3:**
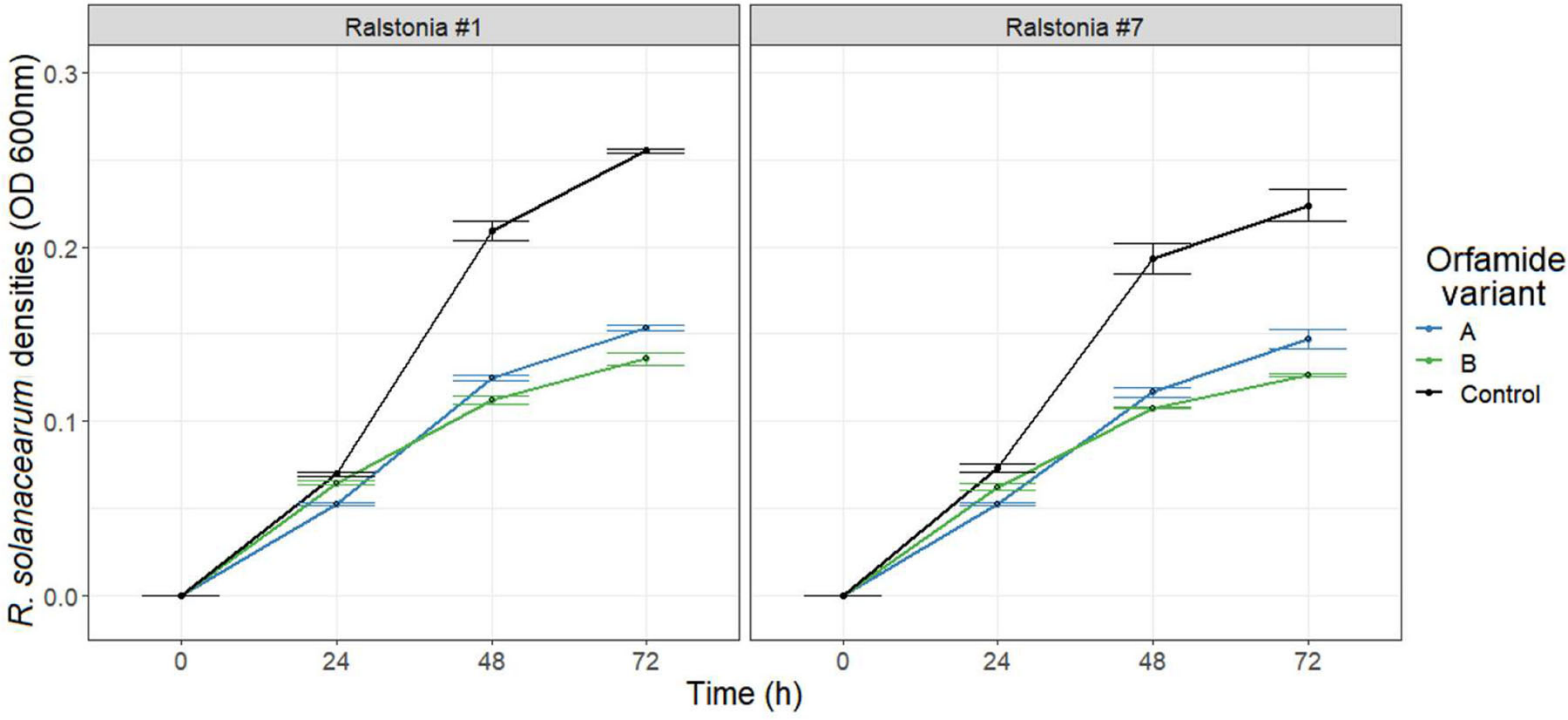
Testing the inhibitory effects of orfamide variants isolated from *Pseudomonas* CHA0 strain on *Ralstonia solanacearum* growth. The black lines denote *R. solanacearum* density in the absence of the orfamide variants over time (control), while colored lines show the growth in the presence of orfamide variants isolated from CHA0. Orfamide variants “A” and “B” refer to two different fractions isolated separately during the extraction process. All error bars show the standard error of the mean (±1 SEM) based on 3 replicates.

Orfamide variants “A” and “B” were isolated from the *Pseudomonas* CHA0 strain and their effects were tested only against *R solanacearum* strains #1 and #7 due to limited quantities of extracted compounds (Figure 3). The growth of both *Ralstonia* strains was constrained by both orfamide variants (*Ralstonia* × *Treatment*: *F*_2, 12_ = 0.0476, *p* = 0.9537, Figure 3) and the growth suppression by the orfamide variants became visible only after 48 h during the assays (*Treatment* × *Time*: *F*_4, 30_ = 75.652, *p* < 0.0001, Figure 3). At the final time point, there were clear differences between orfamide variants on both strain #1 (*Treatment*: *F*_2, 10_ = 681.3, *p* < 0.001, Figure 3) and strain #7 (*Treatment*: *F*_4, 10_ = 65.77, *p* < 0.001, Figure 3), with orfamide variant “B” showing a slightly higher pathogen growth suppression. Together these results suggest that the *Pseudomonas* CHA0 strain produces pathogen‐suppressing orfamide variants in vitro.

### 3.7 Testing *Pseudomonas* CHA0 biocontrol efficacy in vivo in a tomato system

**Figure 4:**
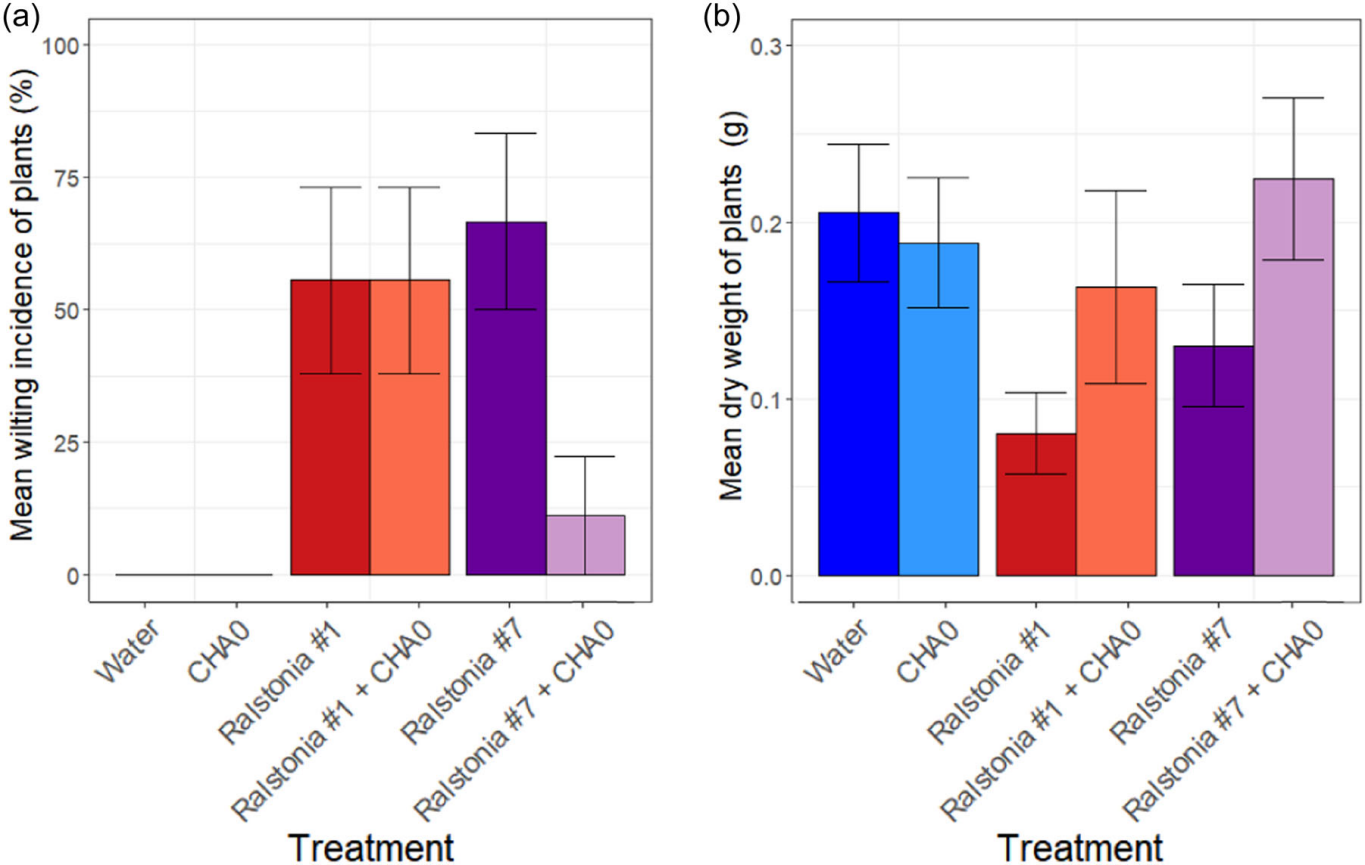
Tomato plant infections by *Ralstonia solanacearum* strains #1 and #7 in the absence and presence of *Pseudomonas* strains. Panel (a) displays the percentage of wilted plants (mean wilting incidence) and panel (b) the mean aboveground dry weight of individual plants at the end of the experiment for all treatments. All error bars show the standard error of the mean (±1 SEM) based on three replicates (every replicate consisting of nine plants)

The biocontrol potential of CHA0 was tested using the Micro‐Tom tomato cultivar infected by the UK *R. solanacearum* strains #1 and #7. In the absence of CHA0, the levels of disease incidence were higher when tomatoes were infected with Strain #7 compared to strain #1 (*Ralstonia: F*_1, 34_ = 48.788, *p* = 0.315, Figure 4a). The CHA0 treatment did not affect wilting incidence compared to the water control treatment in the case of *Ralstonia* strain #1 (*Pseudomonas: F*_1, 16_ = 24.731, *p* = 1, Figure 4a). However, CHA0 reduced wilting incidence of *R. solanacearum* strain #7 (*Pseudomonas: F*_1, 16_ = 17.736, *p* = 0.01114, Figure 4a). The presence of CHA0 also increased the plant dry weight overall (*Pseudomonas: F*_1, 34_ = 4.623, *p* = 0.0387, Figure 4b) and this effect was the same for both *R. solanacearum* strains (*Ralstonia: F*_1, 34_ = 1.668, *p* = 0.205, Figure 4b). Together, these results suggest that CHA0 biocontrol efficacy was dependent on the *R. solanacearum* strain.

## 4 DISCUSSION

This study aimed to screen and identify effective *Pseudomonas* bacterial biocontrol strains against *Ralstonia solanacearum* species complex, understand potential underlying mechanisms of inhibition, and validate their efficacy in vivo in the tomato rhizosphere. *Pseudomonas* strain CHA0 was the most suppressive strain in both direct and indirect *R. solanacearum* inhibition assays. The comparative genomic analysis highlighted that metabolite clusters encoding DAPG, pyoluteorin, and orfamides A and B were unique for the most suppressive CHA0 *Pseudomonas* strain, and while only orfamide A production was detected through mass spectrometry, extracted orfamide variants showed high inhibitory activity against *R. solanacearum*. In vivo tests revealed that CHA0 was effective at reducing bacterial wilt incidence. Interestingly, plant protection depended on the *R. solanacearum* strain identity and was only observed with one of the two tested strains. Despite the successful identification of potential *Pseudomonas* biocontrol species, more work is needed to harness their biocontrol activity in the plant rhizosphere.

When screening for the most effective *Pseudomonas* strains against *R. solanacearum* through direct and indirect assays, *P. protegens* strain CHA0 showed the most suppressive activity against all the tested *R. solanacearum* strains. The strong inhibition effects of CHA0 were observed through both direct and indirect assays. This result is in line with previous studies demonstrating the high biocontrol activity of CHA0 against various plant pathogens (Hu et al., 2016). Although multiple *Pseudomonas* strains have been described in the literature with biocontrol abilities, CHA0 is one of the most well‐established, studied, and successful biocontrol agents against plant‐parasitic nematodes, fungal, and bacterial pathogens (Flury et al., 2017; Humair et al., 2009; Jamali et al., 2009; Jousset et al., 2006; Neidig et al., 2011; Siddiqui & Shaukat, 2003, 2004). Interestingly, some less studied strains, such as MVP1‐4 and Ph11C2, showed also good biocontrol potential and warrant more study in the future. Overall, all *Pseudomonas* strains suppressed all tested *R. solanacearum* strains to some extent in vitro. The likely explanation for this is that European *R. solanacearum* strains are likely to be very similar as they belong to a clonal lineage of Phylotype 2b strains that are adapted to grow in cold climates (Hayward, 1991). In contrast, biocontrol strains belonged to different *P. fluorescens* subgroups (e.g., *P. protegens* and *Pseudomonas corrugata*) and originated from different countries across Europe and America. Hence, *Pseudomonas* strains were likely genetically more dissimilar compared to *R. solanacearum* strains. A more detailed analysis of European *R. solanacearum* strains is however required to link observed phenotypic similarities (equal susceptibility) and differences (infectivity in vivo) with underlying genetic differences.

Comparative genomic analyses revealed that *Pseudomonas* strains CHA0 and Pf‐5 (which belong to the *P. protegens* subgroup of *P. fluorescens*) had the greatest number of secondary metabolite clusters (17), while the other six *Pseudomonas* strains (the majority of which belong to the *P. corrugata* subgroup of *P. fluorescens*) harbored between 11 and 15 metabolic clusters. Based on previous literature, CHA0 and Pf‐5 can produce a similar range of secondary metabolites such as pyoluteorin and cyclic lipopeptides, which could potentially explain why CHA0 exhibited the greatest inhibitory effects (Haas & Keel, 2003; Loper & Gross, 2007; Ma et al., 2016). When testing these candidate metabolites on *R. solanacearum* growth, pyoluteorin stood out as the most suppressive compound, leading to the poorest *R. solanacearum* growth. Only *Pseudomonas* strains CHA0 and Pf‐5 were found to harbor the T1PKS metabolic cluster, which encodes pyoluteorin production. However, no pyoluteorin was produced in vitro by either of the strains in the growth conditions used. Pyoluteorin inactivity in our experiments was potentially due to the use of a rich growth medium (Heidari‐Tajabadi et al., 2011; Jamali et al., 2009; T. Yang et al., 2018). Pyoluteorin is a chlorinated polyketide antibiotic and its production is regulated by the DAPG precursor, monoacetylphloroglucinol (Kidarsa et al., 2011). It is thus possible that efficient production of pyoluteorin limits the production of DAPG, and that these compounds are not produced simultaneously. While CHA0 and Pf‐5 strains harbored the highest amount of NRPS clusters (8 and 7, respectively), they were also associated with a wide variety of other putative metabolites that could have been linked with pathogen suppression (e.g., enantiopyochelin, rhizomide, and pyoverdine). Furthermore, we also isolated uncharacterized orfamide variants from the CHA0 supernatant and observed highly inhibitory effects against the tested *R. solanacearum* strain. This suggests that orfamides at least partly explain the inhibitory capacity of the CHA0 strain in lab conditions. A wealth of other potential antimicrobial compounds was also identified in the CHA0 supernatant using nontargeted analysis. In the future, these secondary metabolites could be isolated and tested in more detailed studies to identify potentially novel antimicrobials (Geudens & Martins, 2018). For example, Rose et al. (2021) have shown that Pf‐5 has notable antialgal properties when interacting with microalga *Chlamydomonas reinhardtii* through the production of rhizoxins and they were also able to detect the production of DAPG, pyrrolnitrin, pyoluteorin, and orfamide A in their experiments. A key difference between ours and their study is that Pf‐5 was grown in a TAP medium under continuous illumination which could have changed the Pf‐5 metabolism and antimicrobial production (Rose et al., 2021). It is thus possible that the use of rich culture media constrained the production of certain secondary metabolites in our assays. In the future, it will be important to characterize the secondary metabolite gene expression and metabolite production across different environments and to identify metabolite production potential in more realistic biocontrol conditions (Deveau et al., 2016; Köhl et al., 2019).

To validate *Pseudomonas* efficacy in vivo, we first tested that both selected *R. solanacearum* strains could infect tomato plants and found that they both caused around 50% bacterial wilt disease incidence. Such variation in disease incidence is typical for *R. solanacearum* as its virulence is determined by a combination of host immunity and environmental conditions that can vary considerably even in controlled greenhouse experiments (Hu et al., 2016; Wei et al., 2019). We found that bacterial wilt incidence was clearly reduced in the presence of CHA0 but only in the case of *R. solanacearum* strain #7. These results contrast with the in vitro results as *R. solanacearum* strains #1 and #7 did not show any differences in their susceptibility to DAPG, pyoluteorin, or orfamide variants produced by CHA0. There are multiple potential biological explanations for this result. For example, it is possible that *Ralstonia* strain #1, was less able to colonize tomato roots, failed to express genes required for protection against *Pseudomonas* inhibition (e.g., efflux pumps or other deactivation of antimicrobials), or was not able to activate virulence gene expression (Ran et al., 2005) in the presence of CHA0. Alternatively, the conditions used in tomato experiments may not have been optimal for *R. solanacearum* survival. Moreover, autoclaving of the soil could have influenced soil properties and microbial metabolism as heating soil over 120°C can result in increased levels of ammonium and nitrogen (Serrasolsas & Khanna, 1995). Together, these findings demonstrate the challenges of translating in vitro results to in vivo applications, highlighting the importance of studying biocontrol effects in more realistic in vivo conditions.

In conclusion, here we show that a combination of in silico, in vitro, and in planta approaches can be used to identify and validate effective biocontrol agents against *R. solanacearum*. By using simple microbiological assays based on direct and indirect interactions with the pathogen we were able to identify *P. protegens* CHA0 as the most inhibitory biocontrol strain. Comparative genomics was used to identify potential secondary metabolite clusters responsible for the inhibition and efficacy of identified compounds and their production was validated in additional experiments. In addition to identified clusters, orfamide variants were also found to be highly inhibitory which could be characterized in the future (Keel et al., 1992; Yasmin et al., 2017). Based on these results, screening for pyoluteorin and orfamide secondary clusters could be used as a rapid way to identify effective biocontrol strains in the future. Such screening methods have been previously used to identify siderophore‐producing biocontrol agents against rice fungal pathogens (Chaiharn et al., 2009). Despite the ability to produce a repertoire of secondary metabolites, CHA0 was able to reduce disease incidence with only one of the two tested *R. solanacearum* strains in vivo. Biocontrol effects should thus be studied in more realistic in vivo conditions in the future to test which mechanisms are expressed and active in the plant rhizosphere and if the natural plant microbiota is also affected by or affects the activity of identified biocontrol bacterial strains. Together, our results suggest that while the developed low‐cost in vitro screening process can be used to identify effective biocontrol agents based on their secondary metabolism, further optimization is needed to predict the function of different strains in the plant rhizosphere.

## AUTHOR CONTRIBUTIONS

**Sophie E. Clough**: Conceptualization (equal); data curation (equal); formal analysis (equal); investigation (equal); methodology (equal); validation (equal); visualization (equal); writing–original draft (equal); writing–review and editing (equal). **Alexandre Jousset**: Data curation (equal); investigation (equal); methodology (equal); writing–review and editing (equal). **John G. Elphinstone**: Investigation (equal); supervision (equal); writing–review and editing (equal). **Ville‐Petri Friman**: Conceptualization (equal); formal analysis (equal); funding acquisition (equal); investigation (equal); project administration (equal); supervision (equal); writing–review and editing (equal).

## CONFLICTS OF INTEREST

None required.

## ETHICS STATEMENT

None required.

## Data Availability

Raw reads for all *Pseudomonas* strains are available at the NCBI Sequence Read Archive under BioProject accession number: PRJNA784243: https://www.ncbi.nlm.nih.gov/bioproject/PRJNA784243.

## 1

See Figure A1, Figure A2, Tables A1 and A2, Table A3, and Tables A4, A5, A6, A7, A8, A9, A10, A11, Table A12.

**Table A1:** Pseudomonas strains used in these experiments, and their geographical origin

*Pseudomonas* strains	*Pseudomonas fluorescens* subgroup	*Pseudomonas* strains' origin
CHA0	(*Pseudomonas protegens*)	Tobacco, Switzerland (Natsch et al., 1994)
Pf‐5	(*P. protegens*)	Cotton, USA (Howell & Stipanovic, 1979)
Q2‐87	(*Pseudomonas corrugata*)	Wheat, USA (Bangera & Thomashow, 1999)
Q8R1‐96	(*P. corrugata*)	Wheat, USA (Raaijmakers & Weller, 1998)
1M1‐96 *(P. fluorescens)*	Not reported	Wheat, USA (Raaijmakers & Weller, 2001)
MVP1‐4 *(P. fluorescens)*	Not reported	Pea, USA (Landa et al., 2002)
F113	(*P. corrugata*)	Sugar beet, Ireland (Shanahan et al., 1992)
Ph11C2 *(P. fluorescens)*	Not reported	Tomato, France (Govaerts et al., 2007)

**Table A2:** *Ralstonia solanacearum* strains used in these experiments, and their geographical origin

*Ralstonia* strains	York sample collection number	*Ralstonia* strains' origin
#1	YO352	River water, UK (2014)
#2	YO354	River water, UK (2015)
#3	YO355	River water, UK (2015)
#4	YO356	River water, UK (2013)
#5	YO162	Commercial culture, Poland (used for verification tests) (2014)
#6	YO351	River water, UK (2013)
#7	YO353	River water, UK (2014)

**Table A3:** Media used for bacterial cultures

Media recipes	
LB broth (1 L)	10 g Tryptone (pancreatic digest of casein)
	5 g Yeast extract
	5 g NaCl
	(+15 g agar for solid media)
CPG broth (1 L)	1 g Casamino acids (casein hydrolysate)
	10 g Peptone
	5 g Glucose
	(+17 g agar for solid media)
NB broth (1 L)	10 g Glucose
	5 g Tryptone
	3 g Beef extract
	0.5 g Yeast extract
Kings Medium B broth (1 L)	20 g Proteose peptone
	1.5 g K_2_HPO_4_
	1.5 g MgSO_4_·7H_2_O
	10 ml Glycerol
Cryomedia for freezing bacterial stocks	50% vol/vol glycerol

**Table A4:** antiSMASH5.0 cluster types recognized in *Pseudomonas* CHA0 genome including the types of clusters and % hit in the database with the most similar known cluster

Cluster	Type	Most similar known cluster
1.1	Bacteriocin	
1.2	CDPS	
1.3	NRPS	Orfamide (94%)
2.1	other	Pyrrolnitrin (100%)
2.2	NRPS	Pyochelin (100%)
2.3	T1PKS	Pyoluteorin (100%)
3.1	NRPS	Lipopeptide (6%)
3.2	NAGGN	
3.3	NRPS	Pyoverdine (16%)
4.1	T3PKS	DAPG (100%)
4.2	Bacteriocin	
5.1	Arylpolyene	APE Vf (40%)
6.1	NRPS	Viscosin (31%) Pyoverdine (14%)
6.2	Betalactone	
8.1	NRPS	Pyoverdine (19%)
10.1	NRPS	Pyoverdine (3%)
13.1	NRPS	

**Table A5:** antiSMASH5.0 cluster types recognized in *Pseudomonas* Pf‐5 genome including the types of clusters and % hit in the database with the most similar known cluster

Cluster	Type	Most similar known cluster
1.1	NRPS	Pyoverdine (14%)
1.2	Betalactone	
1.3	Other	Pyrrolnitrin (100%)
1.4	NRPS	Pyochelin 100%)
1.5	NRPS‐like	Rhizoxin (100%)
1.6	T1PKS	Pyoluteorin (100%)
2.1	Bacteriocin	
2.2	CDPS	
2.3	NRPS	Orfamide (94%)
3.1	NRPS	Pyoverdine (21%)
3.2	NAGGN	
3.3	NRPS	Lipopeptide (6%)
4.1	T3PKS	DAPG (100%)
4.2	Bacteriocin	APE Vf (40%)
5.1	Arylpolyene	
7.1	NRPS	Pyoverdine (19%)
9.1	NRPS	Rhizomide (100%)

**Table A6:** antiSMASH5.0 cluster types recognized in *Pseudomonas* Q2‐87 genome including the types of clusters and % hit in the database with the most similar known cluster

Cluster	Type	Most similar known cluster
1.1	NAGGN	
1.2	NRPS	Pyoverdine (10%)
1.3	Betalactone	
2.1	Butyrolactone	
4.1	T3pks	DAPG (100%)
4.2	Bacteriocin	
5.1	Bacteriocin	
6.1	NRPS	Cupriachelin (35%)
7.1	Arylpolyene	APE vf (40%)
8.1	NRPS	Ishigamide (11%)
9.1	NRPS	Pyoverdine (19%)
9.2	Ectoine	
15.1	Bacteriocin	
19.1	Lanthipeptide	

**Table A7:** antiSMASH5.0 cluster types recognized in *Pseudomonas* Q8R196 genome including the types of clusters and % hit in the database with the most similar known cluster

Cluster	Type	Most similar known cluster
1.1	NRPS	Pyoverdine (10%)
1.2	Betalactone	
1.3	T3PKS	DAPG (100%)
1.4	NRPS	Cupriachelin (11%)
1.5	Butyrolactone	
4.1	Arylpolyene	APE Vf (40%)
6.1	NAGGN	Taiwachelin (11%)
9.1	NRPS	Viscosin (31%) Pyoverdine (10%)
12.1	Bacteriocin	
20.1	NRPS‐Like	Mangotoxin (71%)
23.1	NRPS	Pyoverdine (3%)
24.1	NRPS	Taiwachelin (11%) Pyoverdine (3%)
25.1	NRPS	

**Table A8:** antiSMASH5.0 cluster types recognized in *Pseudomonas* 1M1‐96 genome including the types of clusters and % hit in the database with the most similar known cluster

Cluster	Type	Most similar known cluster
2.1	T3PKS	DAPG (100%)
2.2	NRPS	Cupriachelin (11%)
2.3	Butyrolactone	
3.1	NAGGN	
4.1	NRPS	Serobactin (23%) Pyoverdine (10%)
8.1	Betalactone	
9.1	Bacteriocin	
17.1	Arylpolene	APE Vf (40%)
32.1	Lanthipeptide	Putative class II
44.1	NRPS‐like	Mangotoxin (71%)
49.1	Lanthipeptide	Putative class II
62.1	NRPS	Viscosin (25%) Pyoverdine (6%)

**Table A9:** antiSMASH5.0 cluster types recognized in *Pseudomonas* MVP1‐4 genome including the types of clusters and % hit in the database with the most similar known cluster

Cluster	Type	Most similar known cluster
2.1	Betalactone	
2.2	LAP	
4.1	NRPS	Cupriachelin (11%)
4.2	T3PKS	DAPG (100%)
6.1	Arylpolyene	APE Vf (40%)
9.1	NRPS‐like	Mangotoxin (71%)
10.1	Bacteriocin	
13.1	NAGGN	
24.1	Lanthipeptide	Putative class II
30.1	Butyrolactone	
32.1	NRPS	Viscosin (31%) Pyoverdine (10%)
35.1	NRPS	Pyoverdine (10%)
36.1	NRPS	Pyoverdine (8%), Serobactin (15%), Taiwachelin (11%)

**Table A10:** antiSMASH5.0 cluster types recognized in *Pseudomonas* F113 genome including the types of clusters and % hit in the database with the most similar known cluster

Cluster	Type	Most similar known cluster
2.1	Arylpolyene	APE Vf (40%)
4.1	NAGGN	
4.2	NRPS	Pyoverdine (10%), Serobactins (23%)
5.1	NRPS‐like	Lankacidin (26%)
6.1	Bacteriocin	
11.1	Betalactone	
12.1	T3PKS	DAPG (100%)
16.1	NRPS	Viscosin (31%), Pyoverdine (11%)
19.1	Butyrolactone	
20.1	Lantipeptide	

**Table A11:** antiSMASH5.0 cluster types recognized in *Pseudomonas* Ph11C2 genome including the types of clusters and % hit in the database with the most similar known cluster

Cluster	Type	Most similar known cluster
1.1	NAGGN	
1.2	NRPS	Serobactins (23%) Pyoverdine (10%)
1.3	Betalactone	
2.1	NRPS	
2.2	Bacteriocin	
2.3	Butyrolactone	
4.1	Bacteriocin	
5.1	NRPS‐like	Mangotoxin (71%)
5.2	Bacteriocin	Pyoverdine (1%)
5.3	Arylpolyene	APE Vf (40%)
6.1	NRPS	WLIP (28%), Cupriachelin (11%), Pyoverdine (2%)
6.2	T3PKS	DAPG (100%)
7.1	NRPS	Viscosin (31%), Taiwachelin (27%), Pyoverdine (19%)
12.1	Lanthipeptide	Putative class II

**Table A12:** Putative *Pseudomonas* metabolites produced in LB media identified using Progenesis software

Candidate compound	Metabolite function	Present in CHA0 supernatant	Present in Pf‐5 supernatant
Indole‐3‐acetic acid	Auxin	Yes	Yes
Pyochelin	Siderophore	Yes	
Rhizoxin	Interferes with mitosis by binding to *Beta*‐tubulin affecting microtubule dynamics	Yes	Yes
